# Supplementary material for: Reinforcement learning derived chemotherapeutic schedules for robust patient-specific therapy
Source: Sci Rep. 2021 Sep 9;11:17882. doi: 10.1038/s41598-021-97028-6 (PMC8429726; doi:10.1038/s41598-021-97028-6)
Supplement: Supplementary file 1 — Supplementary Information. [file 41598_2021_97028_MOESM1_ESM.pdf]

Supplementary Information

In Figure S1 we present a visualization of the virtual patients used for testing of all three algorithms and the training virtual patients used for training of the NTNOC algorithm. These figures demonstrate the parameter values chosen via Latin hypercube sampling for the four non-zero bone marrow parameters from Table 1 required to parameterize Eqn. 2.

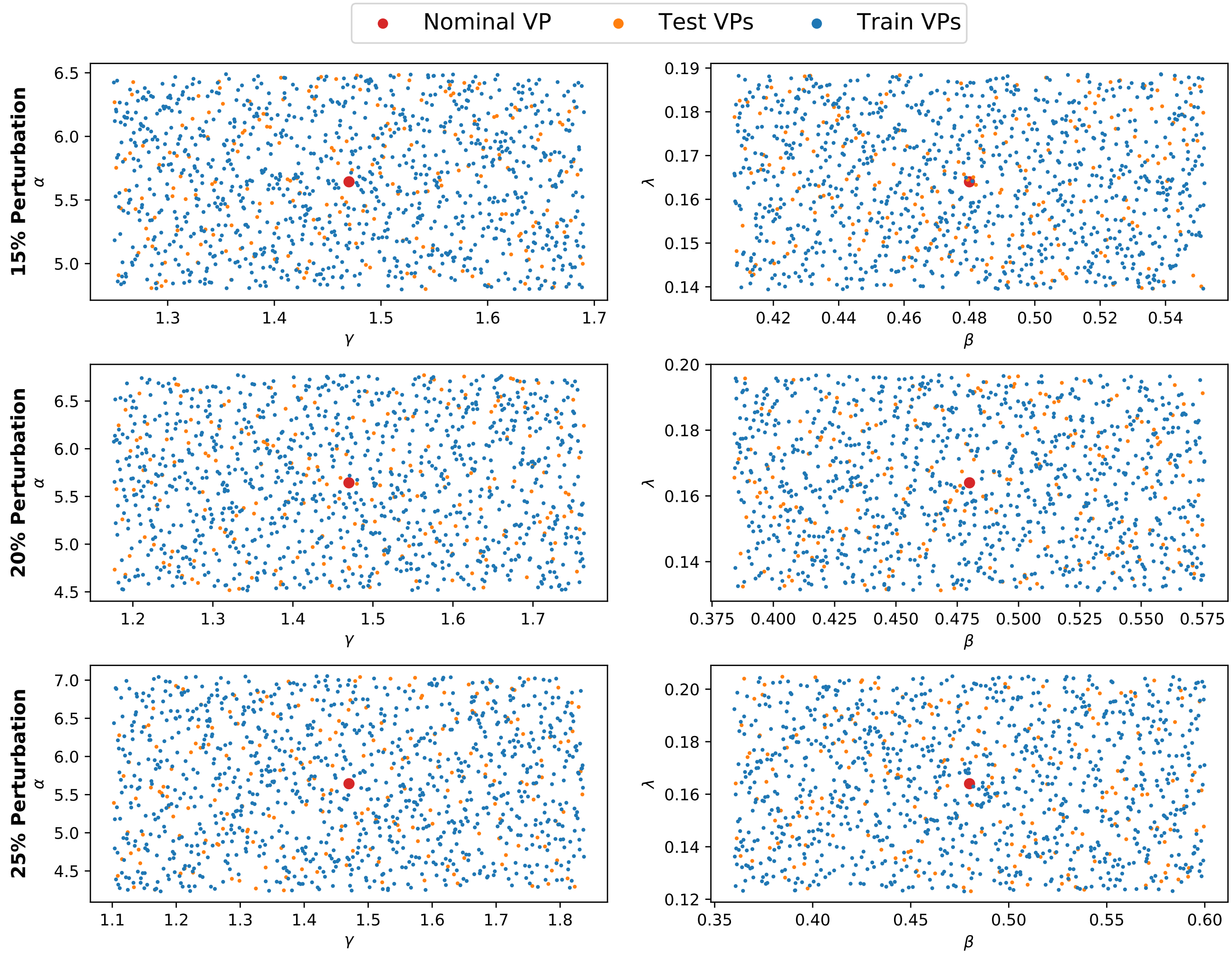

Figure S1. Visualization of the 4 non-zero virtual patient parameters for various perturbation strengths. The orange dots indicate the 200 virtual patients used in testing, the blue dots the 1000virtual patients used in training, and the red dot indicates the location of the nominal virtual patient.

In Figure S2 we present the trajectories of the bone-marrow and the control obtained via the objective functional in Eqn. 3.

In Figure S3 we produce a plot similar to Figure 3 with the proliferative fraction of both the healthy bone-marrow cells and the proliferative fraction of the malignant breast-cancer cells. For this plot, the bone marrow compartments and breast cancer compartments are parameterized with the nominal parameters from Table 1. Notably, in all three scenarios the schedule is able to drive the proliferative fraction of malignant cells down while maintaining the proliferative fraction of the healthy cells. In Figure S3 $\rho_p^{\text{bm}}(t)$ corresponds to the proliferative fraction of the bone-marrow cells and $\rho_p^{\text{bc}}(t)$ corresponds to the proliferative fraction of the breast-cancer cells.

Figure S4 demonstrates various dosing schedules on testing patients. We plot the schedules obtained via employing the reinforcement learning agent trained on the nominal parameter set, the mean optimal controller derived treatment, the NTNOC treatment, and the optimal treatment (which was treated as unknown) acquired by solving the optimal control problem on each particular testing patient (achieved by treating the model parameters as known and employing the APOPT algorithm as implemented in GEKKO[1,2]).

In Figure S5 we present plots of chemotherapy schedules along with the trajectories of the effected bone marrow cells.

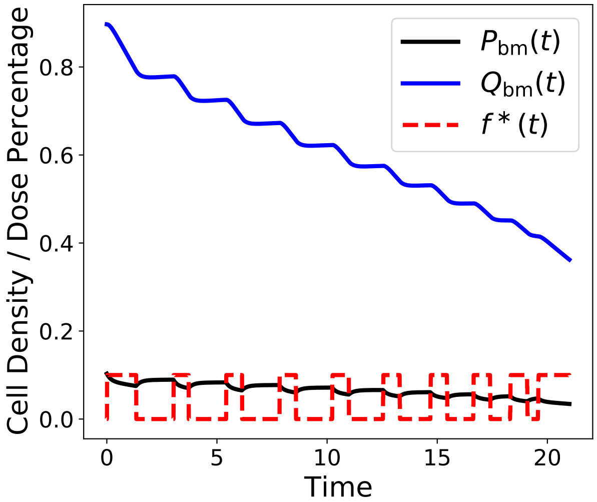

Figure S2. A plot of the Eqn. 1 parameterized for bone-marrow under the optimal control achieved by maximizing the objective functional in Eqn. 3 with $b = 2$. Solved numerically using IPOPT in GEKKO[1,3].

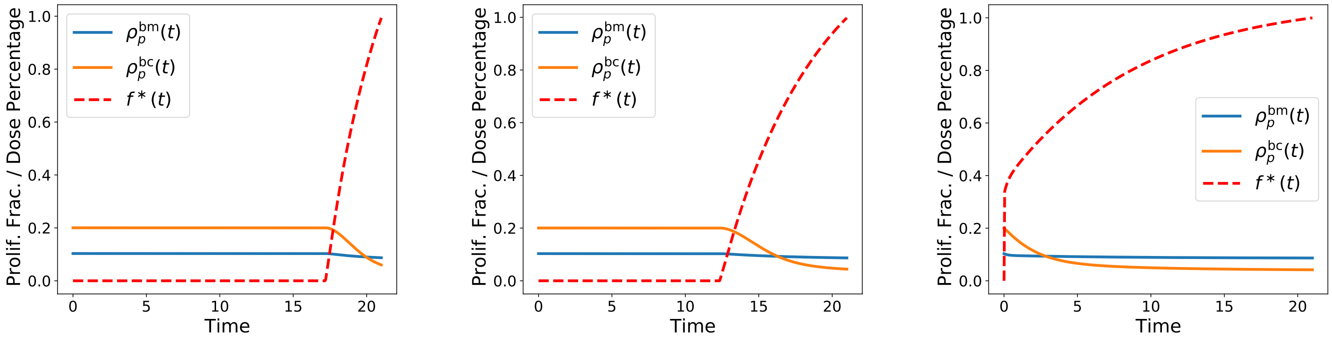

(a) $b = 0.5$ **(b)** $b = 1$ **(c)** $b = 2$

Figure S3. A plot of the proliferative fraction $\rho_p(t) = P(t)/(P(t) + Q(t))$ for both the healthy bone-marrow cells and the malignant breast-cancer cells under the optimal chemotherapeutic control $f^*(t)$ (dashed red) for different values of b. The objective functional used to achieve this optimal control is given via Eq. (2). Small values of b correspond to weighting preservation of the bone marrow as more important and larger values of b correspond to weighting total drug delivery as more important.

These plots are generated for the same virtual patients as in the first column of Figure S4.

The results presented in Figures 7 – 10 all depend upon the particular sample of testing patients (the values of θ_i^k for all k and i). In order to investigate how these results change with differing batches of testing virtual patients, we now present the results of Figure 7 and Figure 9 for five additional batches of 200 testing virtual patients. Explicitly, we independently sampled five batches of 200 testing virtual patients in addition to the batch used in the main manuscript text using the same method as described in the Perturbed Virtual Patients subsection above. For ease of notation, let $B_0^k = \{\theta_i^k \mid 1 \leq i \leq 200\}$ denote the original batch of virtual patients used in the manuscript text at perturbation levels $k \in \{0.15, 0.20, 0.25\}$. Let $B_1^k, B_2^k, \ldots, B_5^k$ represent the 5 additional batches of testing patients sampled independently.

In Figure S6a we present Figure 7b as a probability distribution. Next, in Figures S6b we present the same histogram as displayed as displayed in Figure S6a except $B_1^{0.20}$ was used as the testing set in place of $B_0^{0.20}$. Histograms created with batches $B_2^{0.20}$ to $B_5^{0.20}$ are presented in Figures S6c–S6f. We note the similarities in shape between the histograms presented. In fact, we performed two statistical tests to demonstrate the similarity of the histograms produced by batch $B_0^{0.20}$ with those produced by batches $B_1^{0.20}$ to $B_5^{0.20}$. Explicitly, we considered a two sample Kolmogorov-Smirnov test and a two sample Anderson-Darling test[4,5]. Both tests have the null hypothesis that the two samples are from the same distribution. In all five cases we are unable to reject the null hypothesis ($p > 0.25$).

Similarly, in Figure S7 we present a related visualization. In Figures S7a–S7c we present the results of comparing the

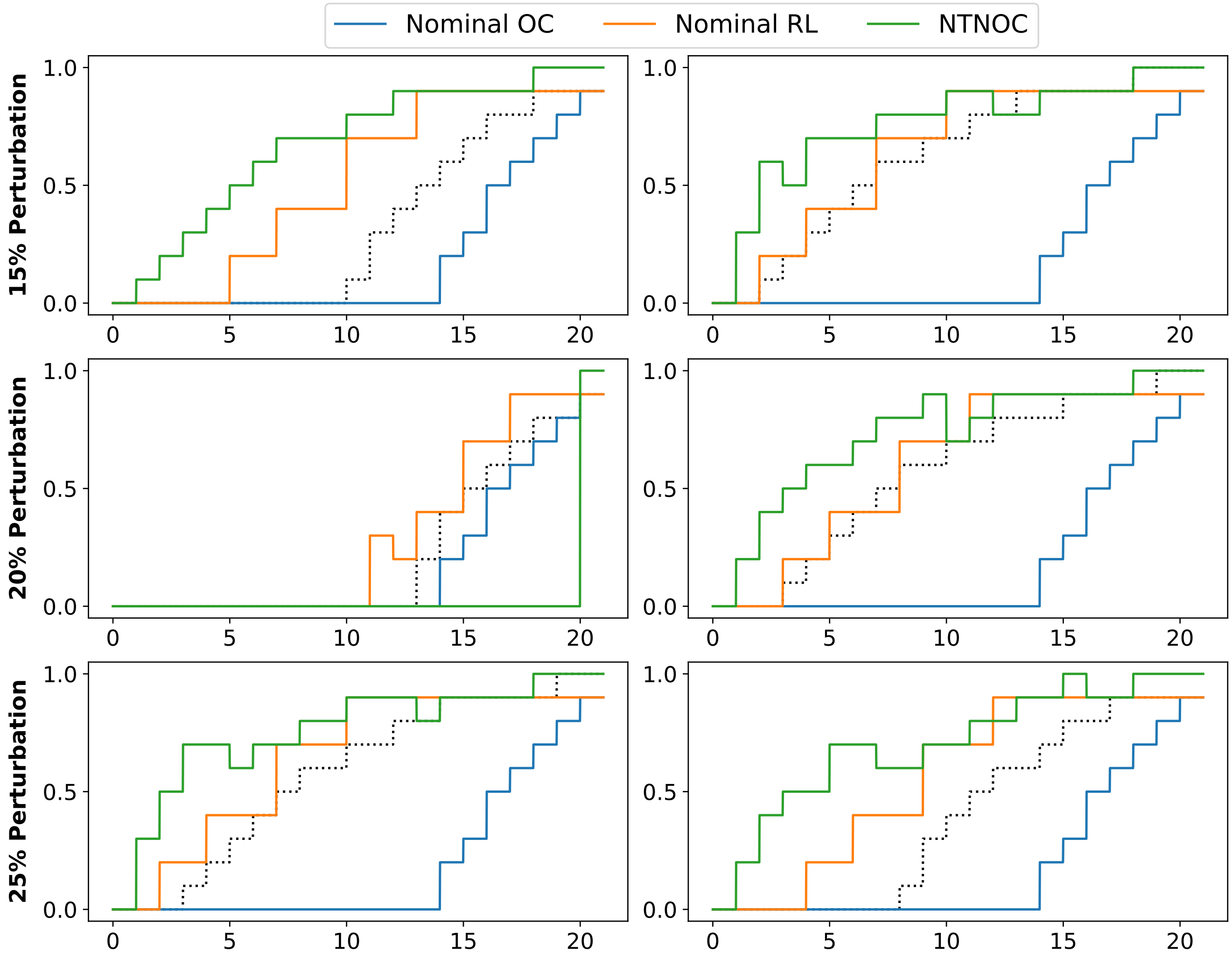

Figure S4. Various chemotherapy dosing schedules obtained on various virtual patients by the three blind methods. In dotted black lines, we also present the theoretically optimal schedule for each patient as a comparison. While the nominal OC schedules remain static (blue lines), the RL and NTNOC schedules adapt to each patient (orange and green lines). In fact, the tendency for the RL schedules (orange lines) to be “closer" (visually) to the theoretical maximal value (dotted lines) visually can demonstrate the tendency for the RL agent to be more robust to perturbations in the parameter values.

reinforcement learner derived treatments from the nominal optimal controller derived treatments on all 6 batches B_0^k to B_5^k at all perturbation strengths. The data for batch B_0^k are presented as solid lines while the data for batches B_1^k to B_5^k are presented as semi-opaque filled histograms stacked on top of one another. This allows one to easily compare the relative shapes of the distribution of these data. In Figures S7d–S7f we present similar data for comparing the reinforcement learner derived treatments with those from the NTNOC derived treatments in the same manner. In all 6 cases, we note that the histograms appear similar to one another via the naked eye. We again applied a two sample Kolmogorov-Smirnov test and a two-sample Anderson-Darling test to formally compare the samples. For all 6 experiments we compared the five new testing batches B_1^k to B_5^k with the reference testing batch B_0^k under the null hypothesis that the two samples are from the same distribution. In all 30 of these such cases we were unable to reject the null hypothesis with either test ($p > 0.25$).

References

1. Beal, L. D., Hill, D. C., Martin, R. A. & Hedengren, J. D. Gekko optimization suite. *Processes* **6**, 106 (2018).
2. Hedengren, J., Mojica, J., Cole, W. & Edgar, T. APOPT: Minlp solver for differential and algebraic systems with benchmark testing. In *Proceedings of the INFORMS National Meeting, Phoenix, AZ, USA*, vol. 1417, 47 (2012).

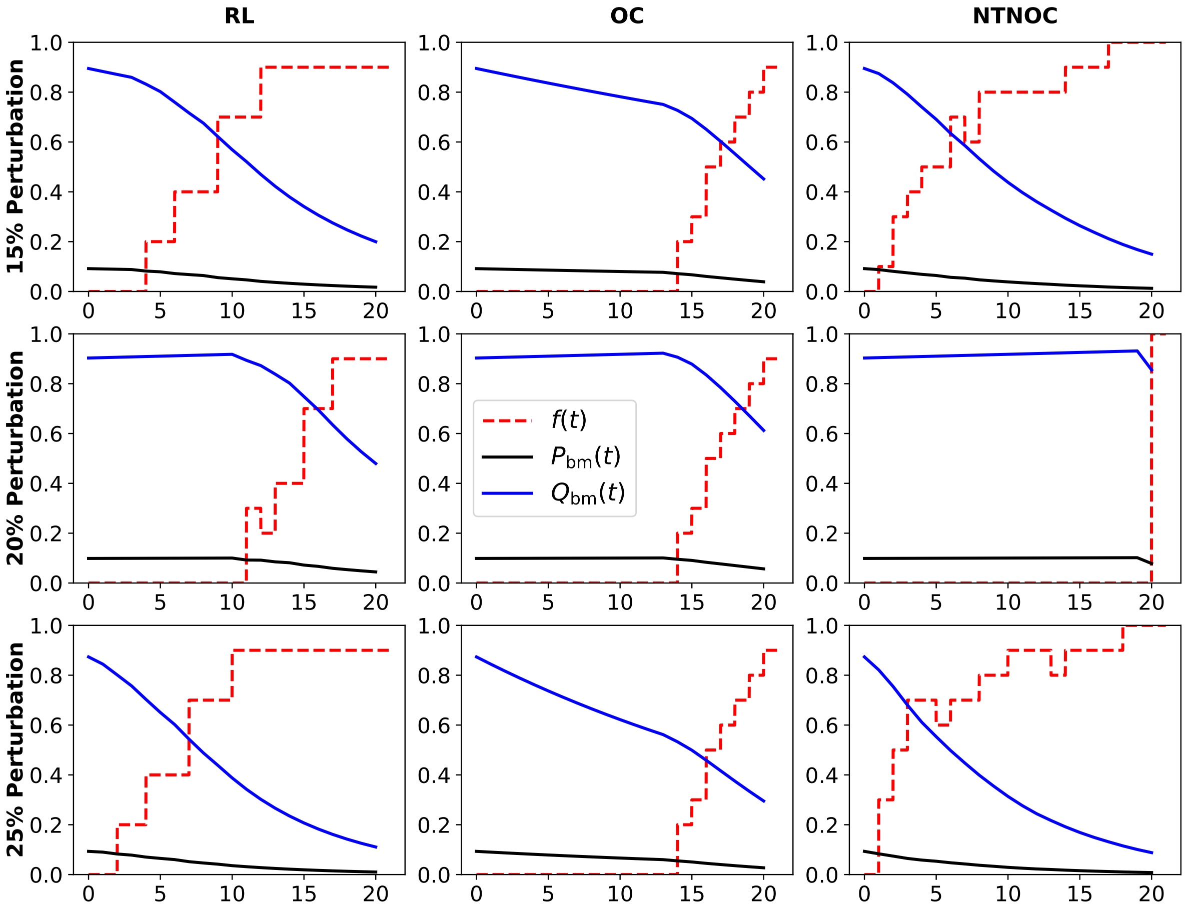

Figure S5. Plots of the proliferative and quiescent bone marrow cells in various virtual patients under chemotherapeutic schedules acquired by the three different learning agents considered in this work.

3. Wächter, A. & Biegler, L. T. On the implementation of an interior-point filter line-search algorithm for large-scale nonlinear programming. *Math. programming* **106**, 25–57 (2006).

4. Hodges, J. L. The significance probability of the Smirnov two-sample test. *Arkiv för Matematik* **3**, 469–486 (1958).

5. Scholz, F. W. & Stephens, M. A. K-sample Anderson–Darling tests. *J. Am. Stat. Assoc.* **82**, 918–924, DOI: 10.1080/01621459.1987.10478517 (1987).

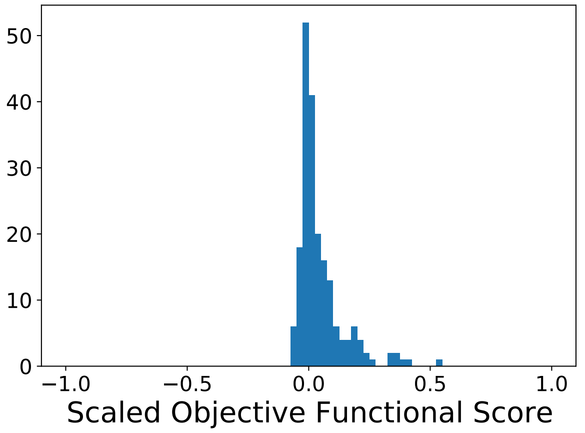

(a) Testing virtual patient batch $B_0^{0.20}$.

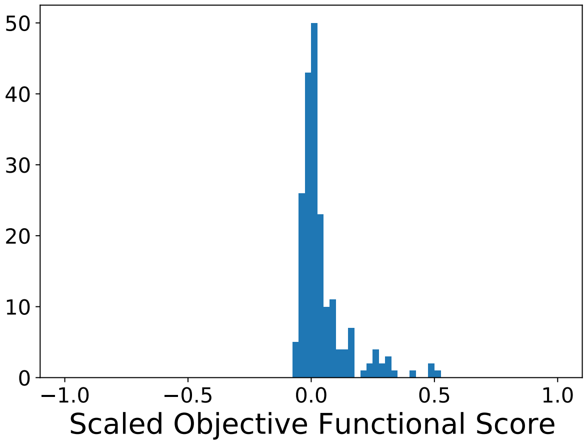

(b) Testing virtual patient batch $B_1^{0.20}$.

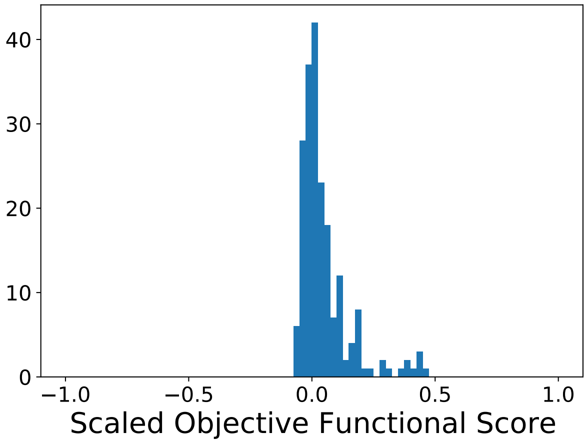

(c) Testing virtual patient batch $B_2^{0.20}$.

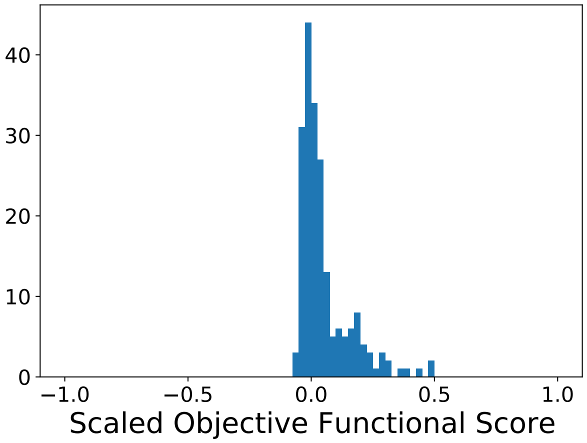

(d) Testing virtual patient batch $B_3^{0.20}$.

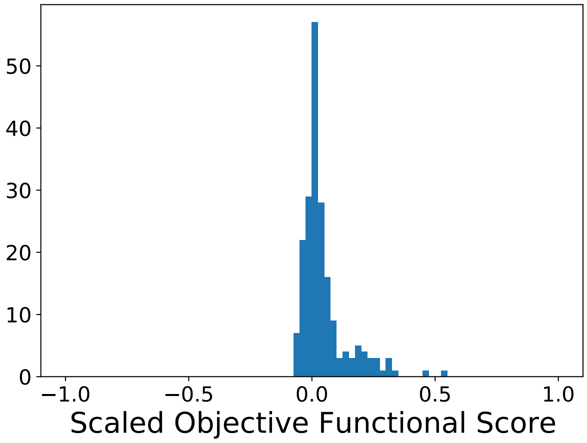

(e) Testing virtual patient batch $B_4^{0.20}$.

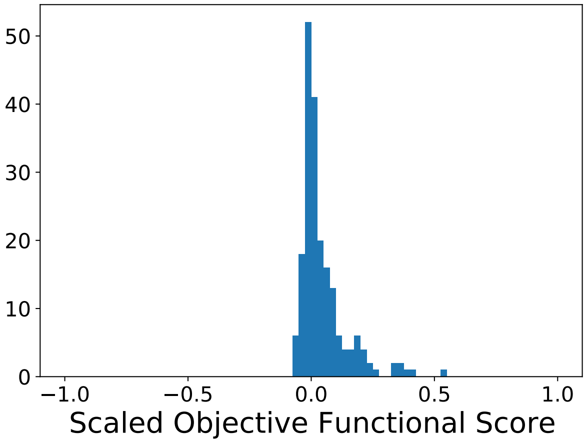

(f) Testing virtual patient batch $B_5^{0.20}$.

Figure S6. Histograms of $\hat{\sigma}_{\mathrm{RL}}(\theta_i^{0.20};\xi_0) - \hat{\sigma}_{\mathrm{OC}}(\theta_i^{0.20};\xi_0)$ where the testing patients θ_i^{20} are from batches $B_0^{0.20}$ to $B_5^{0.20}$. Note the similarly of shapes of the histograms between the reference batch in Figure S6a to the histograms of the independent batches in Figures S6b – S6f.

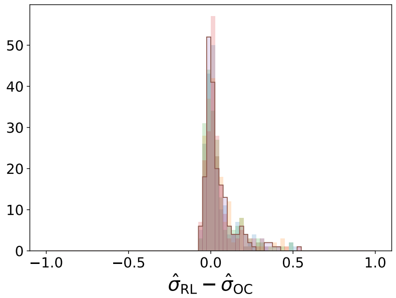

(a) 15% perturbation strength.

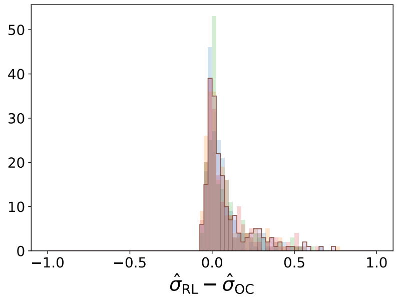

(b) 20% perturbation strength.

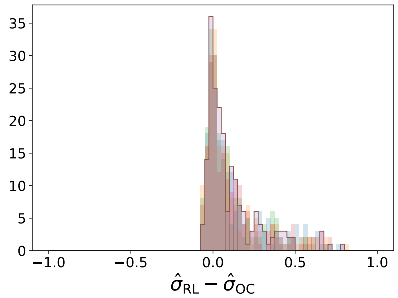

(c) 25% perturbation strength.

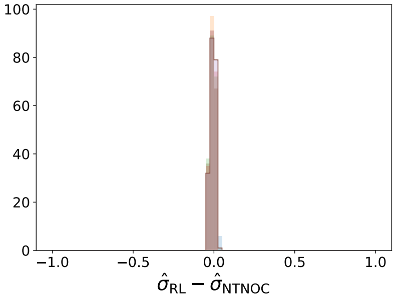

(d) 15% perturbation strength.

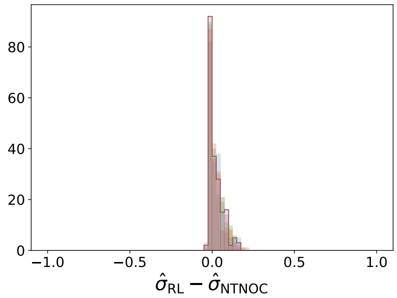

(e) 20% perturbation strength.

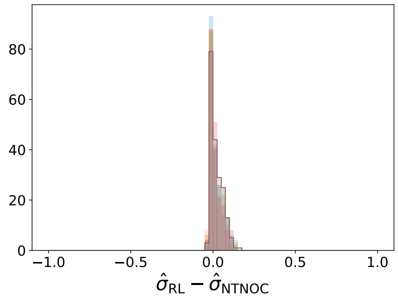

(f) 25% perturbation strength.

Figure S7. A plot comparing the histograms of the difference in scaled objective functional scores. Figures S7a – S7c show histograms of $\hat{\sigma}_{\mathrm{RL}} - \hat{\sigma}_{\mathrm{OC}}$ for various batches while Figures S7d – S7f show histograms of $\hat{\sigma}_{\mathrm{RL}} - \hat{\sigma}_{\mathrm{NTNOC}}$ for various batches. In all plots the histogram of batch B_0^k is represented by the solid outlines and the histograms of batches B_1^k to B_5^k are represented by the semi-opaque filled bars. Neither the Kolmogorov-Smirnov 2-sample test nor the Anderson-Darling 2-sample test could distinguish B_0^k from any of B_1^k to B_5^k.
